# Microbiological Effects of Sodium Hypochlorite/-Amino Acids and Cross-linked Hyaluronic Acid Adjunctive to Non-surgical Periodontal Treatment

**DOI:** 10.3290/j.ohpd.b5281925

**Published:** 2024-04-30

**Authors:** Egle Ramanauskaite, Vita Machiulskiene Visockiene, Yoshinori Shirakata, Anton Friedmann, Laura Pereckaite, Ausra Balciunaite, Urte Marija Dvyliene, Astra Vitkauskiene, Nomeda Baseviciene, Anton Sculean

**Affiliations:** a PhD Student, Clinic of Dental and Oral Pathology, Lithuanian University of Health Sciences, Kaunas, Lithuania. Study design, treatment of patients, data acquisition and interpretation, manuscript writing and revision.; b Professor, Clinic of Dental and Oral Pathology, Lithuanian University of Health Sciences, Kaunas, Lithuania. Manuscript drafting and revision.; c Dentist, Department of Periodontology, Kagoshima University Graduate School of Medical and Dental Sciences, Kagoshima, Japan. Manuscript drafting and revision.; d Professor, School of Dentistry, Faculty of Health, Witten/Herdecke University, Witten, Germany. Manuscript revision.; e PhD Student, Department of Laboratory Medicine, Lithuanian University of Health Sciences, Kaunas, Lithuania. Performed microbiological analysis and interpretation of the data.; f Dentist, Clinic of Dental and Oral Pathology, Lithuanian University of Health Sciences, Kaunas, Lithuania. Manuscript revision, data preparation for statistical analysis, allocation of patients to treatment groups.; g PhD Student, Clinic of Dental and Oral Pathology, Lithuanian University of Health Sciences, Kaunas, Lithuania. Manuscript drafting and revision.; h Professor, Department of Laboratory Medicine, Lithuanian University of Health Sciences, Kaunas, Lithuania. Performed microbiological sampling.; h Professor, Clinic of Dental and Oral Pathology, Lithuanian University of Health Sciences, Kaunas, Lithuania. Manuscript revision.; i Professor and Chair, Department of Periodontology, University of Bern, Bern, Switzerland. Study concept, design and supervision, data interpretation, manuscript writing and revision.

**Keywords:** cross-linked hyaluronic acid, microbiology, non-surgical periodontal therapy, periodontitis, periopathogenic bacteria, sodium hypochlorite/amino acids

## Abstract

**Purpose:**

To investigate the microbiological outcomes obtained with either subgingival debridement (SD) in conjunction with a gel containing sodium hypochlorite and amino acids followed by subsequent application of a cross-linked hyaluronic acid gel (xHyA) gel, or with SD alone.

**Materials and Methods:**

Forty-eight patients diagnosed with stages II-III (grades A/B) generalised periodontitis were randomly treated with either SD (control) or SD plus adjunctive sodium hypochlorite/amino acids and xHyA gel (test). Subgingival plaque samples were collected from the deepest site per quadrant in each patient at baseline and after 3 and 6 months. Pooled sample analysis was performed using a multiplex polymerase chain reaction (PCR)-based method for the identification of detection frequencies and changes in numbers of the following bacteria: *Aggregatibacter actinomycetemcomitans* (A.a), *Porphyromonas gingivalis* (P.g), *Tannerella forsythia* (T.f), *Treponema denticola* (T.d), and *Prevotella intermedia* (P.i).

**Results:**

In terms of detection frequency, in the test group, statistically significant reductions were found for P.g, T.f, T.d and P.i (p < 0.05) after 6 months. In the control group, the detection frequencies of all investigated bacterial species at 6 months were comparable to the baseline values (p > 0.05). The comparison of the test and control groups revealed statistically significant differences in detection frequency for P.g (p = 0.034), T.d (p < 0.01) and P.i (p = 0.02) after 6 months, favouring the test group. Regarding reduction in detection frequency scores, at 6 months, statistically significant differences in favour of the test group were observed for all investigated bacterial species: A.a (p = 0.028), P.g (p = 0.028), T.f (p = 0.004), T.d (p <0.001), and P.i (p = 0.003).

**Conclusions:**

The present microbiological results, which are related to short-term outcomes up to 6 months post-treatment, support the adjunctive subgingival application of sodium hypochlorite/amino acids and xHyA to subgingival debridement in the treatment of periodontitis.

Periodontitis is a chronic, inflammatory disease characterised by microbial dysbiosis, resulting in the destruction of connective tissue attachment and alveolar bone.^1,5,6,16^ Periodontal treatment aims to reduce or eliminate the periodontal-pathogenic biofilm from the periodontal pockets and the surrounding periodontal tissues.^21^ Therefore, the thorough mechanical disruption and removal of subgingival biofilm and calculus are key components of cause-related periodontal therapy, aiming to reestablish clinical health as evidenced by shallow probing depths and the absence of bleeding on probing.^34^

However, the complete removal of plaque and calculus is often limited due to anatomical factors (e.g., furcation involvement, deep pockets, anatomical grooves, or concavities), the operator’s manual skills, and various patient-related factors (e.g., smoking status or systemic diseases). It has been demonstrated that up to 30% of the total surface area of subgingivally debrided roots may still be covered with residual plaque and calculus.^21^ In order to further enhance the elimination of subgingival bacterial biofilm, various adjunctive materials with antimicrobial activity have been utilised.^27^

Recently, a novel concept termed “Clean and Seal” in conjunction with subgingival instrumentation has been suggested to improve the outcomes of non-surgical periodontal therapy.^10,25,26^ The two constituents of “Clean and Seal” are sodium hypochlorite/amino acids (Perisolv, Regedent; Zürich, Switzerland) and cross-linked hyaluronic acid (high molecular) (xHyA) gels (Hyadent BG, Regedent).

Preclinical studies have shown that sodium hypochlorite is able to alter biofilm matrices and act in particular against Gram-negative species associated to periodontitis.^17^ Moreover, favourable cell survival and spreading of periodontal ligament cells has been observed after the application of sodium hypochlorite/amino acids gel to root surfaces.^29^ Clinically, the additive value of sodium hypochlorite/amino acids gel has been reported in treating deep periodontal pockets in untreated periodontitis,^15^ residual periodontal pockets,^18,24^ peri-implant-mucositis^14^ and peri-implantitis.^28^

Preclinical evidence on cross-linked hyaluronic acid has demonstrated that this formulation is not only biocompatible with periodontal tissues but also enhances the proliferative, migratory, and wound healing properties of cells involved in soft-tissue wound healing.^3^ Furthermore, cross-linked hyaluronic acid strongly induces the growth of osteoprogenitors and is able to maintain their stemness, thus potentially regulating the balance between self-renewal and differentiation during bone regeneration.^2^ Importantly, histological evidence from animal studies revealed that the adjunctive application of cross-linked hyaluronic acid resulted in significant regeneration of periodontal tissues in treating intrabony defects, gingival recessions, or furcation defects as compared to surgical controls.^30-32^ Findings from a systematic review have shown that the adjunctive application of hyaluronic acid to non-surgical periodontal treatment resulted in statistically significant improvements in probing depth reduction and gain in clinical attachment compared to controls.^11^

Recent findings from clinical studies have provided evidence indicating that the adjunctive application of sodium hypochlorite/amino acid and cross-linked hyaluronic acid gels to SD may result in significant clinical improvements, as evidenced by the reduction of probing pocket depths (PD), bleeding on probing (BOP), and gain in clinical attachment (CAL). This applies both to patients with untreated periodontitis and patients enrolled in maintenance but still exhibiting residual pockets.^10,25,26^

To the best of our knowledge, no clinical studies to date have reported on the microbiological outcomes following the treatment using this novel concept for non-surgical periodontal therapy. Therefore, the aim of this study was to investigate the potential microbiological advantages of this strategy in the treatment of periodontitis.

## Materials and Methods

### Experimental Design

This randomised, controlled, parallel study included 48 non-smoking patients, diagnosed with stages II-III (grades A, B) generalised periodontitis, aged between 30 to 72 years (mean ± SD), who attended the Department of Dental and Oral Pathology at the Lithuanian University of Health Sciences in Kaunas, Lithuania, for periodontal treatment. The study’s inclusion criteria were the absence of systemic diseases and no intake of medication which may affect periodontal health, the presence of at least 20 teeth, and absence of removable dentures. The study’s exclusion criteria were: smokers, periodontal treatment during last 12 months, antibiotic treatment 3 months prior to the start of the trial, antibiotic prophylaxis required for dental treatment, pregnant/lactating women, and known allergies to sodium hypochlorite. The study protocol was registered at ClinicalTrials.gov, NCT04662216. All patients were enrolled between September 2019 and January 2022. Each patient was given detailed information of the study protocol and was required to sign an informed consent form.

### Treatment Procedures

**Fig 1:**
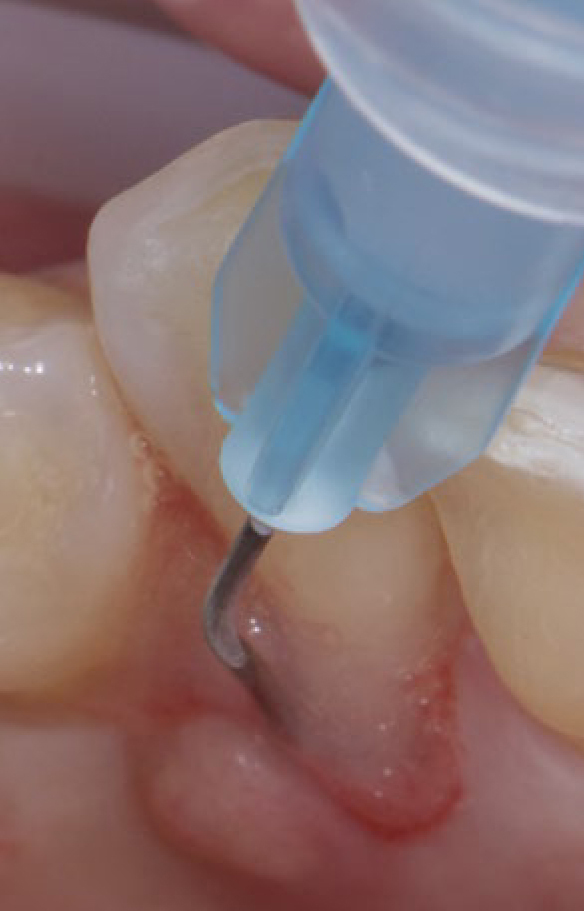
The application of sodium hypochlorite/ amino acid gel to the periodontal pocket prior to subgingival debridement.

**Fig 2:**
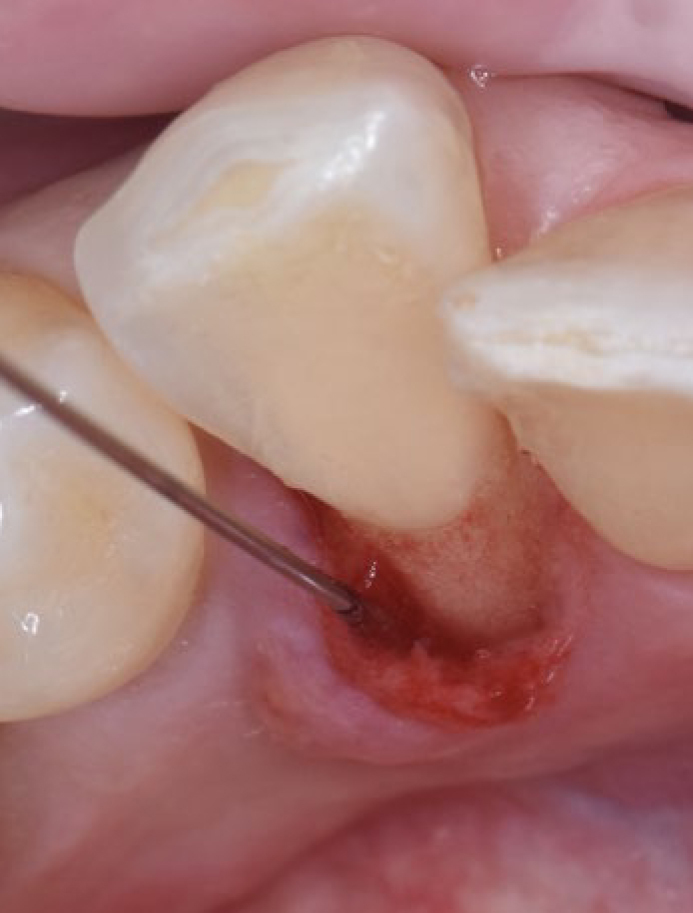
Application of a mixture of natural and cross-linked hyaluronic acid (high molecular) to the periodontal pocket after subgingival debridement.

After an initial screening visit for recruitment and supragingival cleaning, patients were assigned randomly to the control or test groups (control group: 24 patients; test group: 24 patients). Demographic details, randomisation, allocation concealment and study design are described in detail in a related paper reporting clinical outcomes.^26^ In brief, subjects in the control group underwent full-mouth SD performed with ultrasonic (Satelec/Acteon suprasson newtron ultrasonic scaler, Acteon; Norwich, UK) and hand instruments (LM SharpDiamond 1/2, 7/8, 11/12, 13/14 SD mini Gracey and Gracey curettes, LM; Parainen, Finland). Subsequently, all teeth were polished using a low-abrasive paste (Lunos Super Soft, RDA < 5, Dürr Dental; Bietigheim-Bissingen, Germany). In the test group, full-mouth SD was performed as follows: in all pockets with PD ≥ 4 mm, a sodium hypochlorite/amino acid gel (Perisolv, Regedent) was inserted into the pockets and left there for 60 s before subgingival instrumentation (Fig 1). Subgingival instrumentation was carried out with the same ultrasonic and hand instruments, and the application of sodium hypochlorite/amino acid gel was repeated until instrumentation was considered sufficient (i.e., a total of 2–3 times). Following SD and polishing, a mixture of natural and cross-linked hyaluronic acid (high molecular) gel (Hyadent BG, Regedent) was inserted in the pockets using a blunt needle (Fig 2).

### Outcomes

The primary outcome variable was the change in detection frequency of *Aggregatibacter actinomycetemcomitans* (A.a), *Porphyromonas gingivalis* (P.g), *Tannerella forsythia* (T.f), *Treponema denticola* (T.d), and *Prevotella intermedia* (P.i) from baseline to 6 months. Secondary outcome variables included the change of detection scores (0-4, which correspond to the number CFUs, see Table 1) of the respective bacteria as well as changes in PD, CAL, BOP and plaque index (PI) at sampled sites from baseline to 6 months.

**Table 1:** Semi-quantitative interpretation of the test results

Bacterial species	Color intensity of the test strip bands representing detection scores 0-4				
	0	1	2	3	4
*Aggregatibacter actinomycetemcomitans*	<10^3^ CFU/ml	10^3^ CFU/ml	<10^4^ CFU/ml	<10^5^ CFU/ml	>10^6^ CFU/ml
*Porphyromonas gingivalis*	<10^4^ CFU/ml	10^4^ CFU/ml	<10^5^ CFU/ml	<10^6^ CFU/ml	>10^7^ CFU/ml
*Prevotella intermedia*					
*Tannerella forsythia*					
*Treponema denticola*					

### Microbial Sampling

Subgingival plaque samples were collected at baseline (prior to SD) and at 3 and 6 months from the deepest pocket per quadrant by the same investigator (U.M.D). Following a thorough removal of supragingival plaque and calculus using periodontal curettes and sterile cotton pellets, each site was dried and isolated with cotton rolls. A sterile endodontic paper point ISO #30 (Dentsply Sirona; Bensheim, Germany) was inserted and left in place for 20 s. Four samples per patient were collected in a coded sterile-sealed Eppendorf tube and sent to the laboratory (Department of Laboratory Medicine, Lithuanian University of Health Sciences, Kaunas, Lithuania) for analysis. There, these samples were kept frozen at -20°C until further processing (for one day), and then at -80°C until the microbiological analysis was performed (not more than 30 days later). Molecular analysis of the subgingival plaque samples was performed manually in three steps:

- deoxyribonucleic acid (DNA) extraction;
- multiplex amplification with biotinylated primers;
- reverse hybridisation.

### DNA Extraction

DNA extraction was performed using DNA purification from swab samples kit (Swab, version 0517, A&A Biotechnology; Gdynia, Poland). 700 µl of lysis solution and 20 µl of proteinase K were added to the original Eppendorf tubes containing the paper points with subgingival plaque samples. The tube contents were thoroughly mixed, briefly centrifuged, and incubated for 20 min at 37°C with mixing at 500 rpm. After incubation, the samples were mixed, centrifuged, and the resulting liquid was applied to the spin columns. The columns were centrifuged for 1 min at 12,000 rpm. Two washing cycles were performed using new 2-ml tubes and 500 µl of washing solution each time. The washing solution was centrifuged at 12,000 rpm for 1 min the first time and for 2 min the second time. The washed and spun columns were transferred to new 1.5-ml tubes, and 150 µl of elution buffer heated to 75°C was added, incubated for 3 min at room temperature, and centrifuged for 1 min at 12,000 rpm. The resulting DNA samples were stored at -80°C until further analysis.

### Multiplex DNA Amplification

DNA samples were analysed using molecular genetic assay for combined identification of five periodontopathogenic bacterial species (micro-IDent VER 2.0, Hain Lifescience; Nehren, Germany) including *Aggregatibacter actinomycetemcomitans, Porphyromonas gingivalis, Prevotella intermedia, Tannerella forsythia,* and *Treponema denticola*. Master mix of the amplification enzymes was freshly prepared before testing each batch of the DNA samples. 45 µl of master mix and 5 µl of DNA samples or the negative control (molecular-biology–grade water) were prepared and mixed in separately designated laboratory spaces. The negative control was used along with each 24-sample batch. Polymerase chain reaction (PCR) for DNA amplification was performed in the thermal cycler according to the protocol provided by the diagnostic kit’s manufacturer. Amplification products were stored at 2-4°C until further processing.

### Reverse Hybridisation

Before starting the test procedure, as stated in the manufacturer’s instructions, reagents were brought to room temperature (20-25°C) or heated to 45°C, and necessary dilutions were prepared. First, 20 µl of denaturation solution and 20 µl of amplified DNA sample were mixed and incubated at room temperature for 5 min. 1 ml of pre-warmed hybridisation buffer was added, and test strips were placed into each well containing denatured DNA samples. The prepared wells were incubated for 30 min at 45°C in a shaking water bath. After incubation, the hybridisation buffer was aspirated, and 1 ml of stringent wash solution was added to each well. The wells were incubated for 15 min at 45°C in the shaking water bath. The stringent wash solution was removed, and each strip was washed with 1 ml of rinse solution for 1 min on a shaking platform. 1 ml of diluted conjugate solution was added to each well and incubated for 30 min at room temperature on the shaking platform. Conjugate was removed, and each strip was washed for 1 min three times on a shaking platform: twice using rinse solution and once using distilled water. 1 ml of diluted substrate solution was added to each well and incubated protected from light and without shaking for 15 min. After test strip bands became clearly visible, they were briefly washed twice using distilled water, dried between two layers of absorbent paper, pasted on the provided evaluation sheet and stored protected from light.

### Evaluation and Interpretation of Microbiological Results

First, developed test strips were inspected for effective and correct testing procedure by observing three control bands (conjugate control, hybridisation control, and amplification control). After making sure all three control bands were correctly developed, five bacterial species-specific bands were analysed by a semi-quantitative approach. According to the developed color intensity, 0, 1, 2, 3, or 4 points were assigned to each band. The color intensity of the bands is expressed semi-quantitatively as detection scores 0-4, which represent the number of CFUs/ml (Table 1).

### Clinical Measurements

The following clinical parameters were measured to the nearest mm using a Williams periodontal probe (LM 51 ES, LM-Dental; Parainen, Finland) from the deepest site per quadrant at baseline, 3 and 6 months following the treatment:

- Bleeding on probing (BOP), defined as the percentage of sites positive for bleeding within 10 s after probing (%).
- Plaque index (PI), defined as the percentage of sites with visual plaque on the tooth surface (%).
- Probing depth (PD), measured in mm from the gingival margin to the bottom of the probed pocket.
- Recession (REC), measured in mm from the gingival margin to the cementoenamel junction or to the margin of a cervical restoration.
- Clinical attachment level (CAL), calculated by adding PD and REC at each site.

### Blinding

Clinical measurements and microbial sampling were performed by a blinded calibrated examiner (U.M.D.), who was not aware in any of the cases of the type of treatment performed. To ensure blindness, the treatment procedures were performed by one experienced periodontist (E.R.). A third investigator (L.P.) performed microbiological analysis and was unaware of neither treatment procedures nor clinical measurements. I.N. processed coded data for statistical analysis.

### Statistical Analysis

Statistical analysis was performed with the IBM SPSS 27 software package (IBM; Armonk, NY, USA). Data analysis was performed using the patient as the statistical unit. The difference in the distribution of sampled sites in terms of tooth group was examined using the Mann-Whitney U-test for two independent groups. For clinical changes at sampled sites, mean values per subject and per visit were calculated for each clinical parameter. The Shapiro-Wilk test was performed to assess whether clinical periodontal measures followed a normal distribution. If data followed a normal distribution, a paired-samples t-test was performed to evaluate before- and after-treatment comparisons within groups. If the data did not follow a normal distribution, the Wilcoxon signed-rank test was performed on related samples to assess before- and after-treatment comparisons within the groups. The between-group comparisons of measures were obtained by either the independent-samples t-test (if a parameter followed a normal distribution) or the Mann-Whitney test (if a specific measure followed a non-normal distribution).

Differences in detection frequency (0 = undetected and 1 = detected) between the control group and the test group at baseline and at 3 and 6 months were analysed using the Χ^2^ test. The within-group changes were evaluated by McNemar test.

The changes of the detection frequency scores were recorded and classified into one of the following categories: 0: not detectable; or detectable with a score of 1, 2, 3 or 4 (Table 1). Intragroup comparisons of detection scores of periopathogen species between the baseline and 3- and 6-month evaluation were performed using the Wilcoxon signed-rank test. The Mann-Whitney test was used for intergroup comparisons of detection scores for each timepoint. The significance level was set at 0.05.

## Results

All 48 patients completed the study. The distribution of sampled sites was equal in both groups in terms of tooth group, except for lateral incisors (Table 2).

**Table 2:** Distribution of sampled sites

Treatment	Second molars	First molars	Second premolars	First premolars	Canines	Lateral incisors	Central incisors
Control group (n)	10	5	9	10	16	25	21
Test group (n)	9	9	14	11	21	13	19
p	0.621	0.244	0.503	0.152	0.327	0.021	0.504

Mann-Whitney U-test for two independent groups.

### Detection Frequency of Periodontopathogens

Table 3 displays the detection frequencies for each periodontopathogen at different time points in test and control groups. The results were expressed as the proportion of patients (%) positive for a given pathogen.

**Table 3:** Detection frequencies sorted by periodontopathogen (%)

Periodontopopathogen	Treatment strategy	Baseline	3 months	6 months
Aa	Control group	42.5	54.2	58.3
	Test group	45.8	29.2	33.3
Pg	Control group	75.0	58.3	75.0*
	Test group	87.5^a^^b^	41.7^a^	41.7*^b^
Tf	Control group	91.7^a^	62.5^a^	79.2
	Test group	83.3^a^^b^	54.2^a^	58.3^b^
Td	Control group	87.5^a^	58.3^a^	79.2*
	Test group	95.8^a^^b^	41.7^a^	33.3*^b^
Pi	Control group	58.3	29.2	45.8*
	Test group	45.8^a^^b^	20.8^a^	8.3*^b^

*p < 0.05, between-group differences (Χ^2^ test). ^a^p < 0.05, baseline and after 3 months (McNemar test); ^b^p < 0.05, baseline and after 6 months (McNemar test).

In the control group, after 3 months, statistically significant reductions were detected for T.f and T.d (p < 0.05), whereas after 6 months, the detected frequencies of the respective bacteria recovered to pretreatment levels and were comparable to the baseline values (p > 0.05). In the test group, statistically significant reductions were found for for P.g, T.f, T.d and P.i after 3 and 6 months (p < 0.05). The comparison of the test and control groups pointed to statistically significant differences in detection frequency of P.g (p = 0.034), T.d (p < 0.01) and P.i (p = 0.02) after 6 months, favouring the test group.

### Changes of the Detection Scores of Periodontopathogens

Table 4 shows detection scores for A.a, P.g, T.f, T.d, and P.i at baseline, 3- and 6-month follow-ups.

**Table 4:** Detection frequency scores for A.a, P.g, P.i, T.f, T.d at baseline, 3- and 6-month follow-up visits

Species	Timepoint	Detection score	Total, n (%)	Control group, n (%)	Test group, n (%)	p–value**
A.a	Baseline	0 1 2 3 4	22 (45.8) 2 (4.2) 4 (8.3) 8 (16.7) 12 (25.0)	9 (37.5) 1 (4.2) 2 (8.3) 4 (16.7) 8 (33.3)	13 (54.2) 1 (4.2) 2 (8.3) 4 (16.7) 4 (16.7)	0.174
	3 months	0 1 2 3 4	28 (58.3) 2 (4.2) 6 (12.5) 6 (12.5) 6 (12.5)	11 (45.8) 1 (4.2) 4 (16.7) 2 (8.3) 6 (25.0)	17 (70.8) 1 (4.2) 2 (8.3) 4 (16.7) 0	0.044
	*p–value		0.013	0.231	0.011	
	6 months	0 1 2 3 4	26 (54.2) 4 (8.3) 4 (8.3) 6 (12.5) 8 (16.7)	10 (41.7) 1 (4.2) 3 (12.5) 3 (12.5) 7 (29.2)	16 (66.7) 3 (12.5) 1 (4.2) 3 (12.5) 1 (4.2)	0.028
	*p–value		0.085	0.064	0.016	
P.g	Baseline	0 1 2 3 4	9 (18.8) 1 (2.1) 2 (4.2) 11 (22.9) 25 (52.1)	6 (25.0) – 1 (4.2) 4 (16.7) 13 (54.2)	3 (12.5) 1 (4.2) 1 (4.2) 7 (29.2) 12 (50.0)	0.884
	3 months	0 1 2 3 4	24 (50.0) 3 (6.3) 6 (12.5) 8 (16.7) 7 (14.6)	10 (41.7) 1 (4.2) 3 (12.5) 4 (16.7) 6 (25.0)	14 (58.3) 2 (8.3) 3 (12.5) 4 (16.7) 1 (4.2)	0.099
	*p–value		<0.001	0.013	<0.001	
	6 months	0 1 2 3 4	20 (41.7) 7 (14.6) 8 (16.7) 6 (12.5) 7 (14.6)	6 (25.0) 4 (16.7) 4 (16.7) 3 (12.5) 7 (29.2)	14 (58.3) 3 (12.5) 4 (16.7) 3 (12.5) –	0.006
	*p–value		<0.001	0.039	<0.001	
T.f	Baseline	0 1 2 3 4	6 (12.5) 3 (6.3) 5 (10.4) 18 (37.5) 16 (33.3)	2 (8.3) 3 (12.5) 3 (12.5) 8 (33.3) 8 (33.3)	4 (16.7) – 2 (8.3) 10 (41.7) 8 (33.3)	0.846
	3 months	0 1 2 3 4	20 (41.7) 8 (16.7) 8 (16.7) 10 (20.8) 2 (4.2)	9 (37.5) 1 (4.2) 5 (20.8) 7 (29.2) 2 (8.3)	11 (45.8) 7 (29.2) 3 (12.5) 3 (12.5) –	0.088
	*p–value		<0.001	0.007	<0.001	
	6 months	0 1 2 3 4	15 (31.3) 10 (20.8) 9 (18.8) 12 (25.0) 2 (4.2)	5 (20.8) 3 (12.5) 4 (16.7) 10 (41.7) 2 (8.3)	10 (41.7) 7 (29.2) 5 (20.8) 2 (4.2) –	0.004
	*p–value		<0.001	0.048	<0.001	
T.d	Baseline	0 1 2 3 4	4 (8.3) 10 (20.8) 22 (45.8) 12 (25.0) –	3 (12.5) 4 (16.7) 11 (45.8) 6 (25.0) –	1 (4.2) 6 (25.0) 11 (45.8) 6 (25.0) –	0.878
	3 months	0 1 2 3 4	24 (50.0) 13 (27.1) 10 (20.8) 1 (2.1) –	10 (41.7) 6 (25.0) 7 (29.2) 1 (4.2) –	14 (58.2) 7 (29.2) 3 (12.5) – –	0.125
	*p–value		<0.001	0.003	<0.001	
	6 months	0 1 2 3 4	21 (43.8) 13 (27.1) 12 (25.0) 2 (4.2) –	5 (20.8) 6 (25.0) 11 (45.8) 2 (8.3) –	16 (66.7) 7 (29.2) 1 (4.2) – –	<0.001
	*p–value		<0.001	0.083	<0.001	
P.i	Baseline	0 1 2 3 4	23 (47.9) 5 (10.4) 4 (8.3) 12 (25.0) 4 (8.3)	10 (41.7) 4 (16.7) – 8 (33.3) 2 (8.3)	13 (54.2) 1 (4.2) 4 (16.7) 4 (16.7) 2 (8.3)	0.413
	3 months	0 1 2 3 4	36 (75.0) 2 (4.2) 7 (14.6) 3 (6.3) –	17 (70.8) 1 (4.2) 3 (12.5) 3 (12.5) –	19 (79.2) 1 (4.2) 4 (16.7) – –	0.399
	*p–value		<0.001	0.012	0.014	
	6 months	0 1 2 3 4	35 (72.9) 4 (8.3) 5 (10.4) 4 (8.3) –	13 (54.2) 3 (12.5) 4 (16.7) 4 (16.7) –	22 (91.7) 1 (4.2) 1 (4.2) – –	0.003
	*p–value		<0.001	0.091	0.003	

n: frequencies; *according to Wilcoxon tests for intragroup comparison of pathogen detection scores between successive timepoints; **according to Mann–Whitney tests for intergroup comparisons of pathogen detection scores for each timepoint.

At baseline, no statistically significant differences were observed between control and test groups in terms of detection scores of the investigated periodontal pathogenic species (p > 0.05). In the control group at 3 months, a statistically significant decrease in detection scores from baseline was found for P.g (p = 0.013), T.f (p = 0.007), T.d (p = 0.003) and P.i (p = 0.012). At 6 months, statistically significant reductions from baseline remained for P.g (p = 0.039) and T.f (p = 0.048). The test group at 3 months demonstrated a statistically significant decrease in detection scores from baseline for all investigated periopathogenic species: A.a (p = 0.011), P.g (p < 0.001), T.f (p < 0.001), T.d (p < 0.001) and P.i (p = 0.014). These results were maintained after 6 months: A.a (p = 0.016), P.g (p < 0.001), T.f (p < 0.001), T.d (p < 0.001) and P.i (p = 0.003). The intergroup analysis exhibited statistically significant differences in detection scores between control and test groups for A.a (p = 0.044) at the 3-month evaluation and for A.a (p = 0.028), P.g (p = 0.006), T.f (p = 0.004), T.d (p < 0.001) and P.i (p = 0.003) at the 6-month evaluation, favouring the test group.

### Clinical Changes at Sampled Sites

Clinical changes at sampled sites are depicted in Table 5.

**Table 5:** Clinical data of sampled sites (mean ± SD) at different time points

PD (mm)	Control group	Test group	p-value
		
Baseline	6.4 (1.0)	6.6 (1.2)	0.569^a^
After 3 months	3.3 (1.0)	2.5 (0.9)	0.02^a^
Baseline vs 3 months	<0.001^b^	<0.001^b^	
After 6 months	3.6 (0.8)	2.0 (0.8)	<0.001^a^
Baseline vs 6 months	<0.001^b^	<0.001^b^	
3 months vs 6 months	0.096^b^	0.003^b^	
CAL (mm)			
Baseline	6.4 (1.2)	6.4 (1.4)	0.844^a^
After 3 months	3.5 (1.0)	2.8 (1.2)	0.017^a^
Baseline vs 3 months	<0.001^b^	<0.001^b^	
After 6 months	3.9 (1.0)	2.3 (1.1)	<0.001^a^
Baseline vs 6 months	<0.001^b^	<0.001^b^	
3 months vs 6 months	0.084^b^	0.003^b^	
BOP (%)			
Baseline	92.1 (5.9)	94.2 (4.4)	0.429^a^
After 3 months	52.1 (14.2)	32.2 (14.6)	0.003^a^
Baseline vs 3 months	<0.001^b^	<0.001^b^	
After 6 months	59.4 (16.2)	19.2 (11.2)	<0.001^a^
Baseline vs 6 months	<0.001^b^	<0.001^b^	
3 months vs 6 months	0.072^b^	0.002^b^	
PI (%)			
Baseline	66.2 (22.1)	68.2 (11.2)	0.622^a^
After 3 months	21.2 (17.1)	19.2 (11.2)	0.002^a^
Baseline vs 3 months	<0.001^b^	<0.001b	
After 6 months	26.2 (21.3)	13.3 (6.8)	0.006^a^
Baseline vs 6 months	0.041^b^	<0.001^b^	
3 months vs 6 months	0.062^b^	0.003^b^	

^a^ Statistical analysis using the Mann-Whitney test for two independent groups. ^b^ Paired-samples t-test for two dependent groups.

At baseline, no statistically significant differences were observed between test and control groups in any of the investigated clinical parameters (p > 0.05).

Regarding PD changes, both groups demonstrated statistically significant reductions in PD after 3 and 6 months; however, the difference between groups was statistically significant in favour of the test group at both timepoints (p = 0.02 and p < 0.001, respectively). Importantly, the PD change between 3- and 6- month follow-ups was statistically significant in the test group (p = 0.003), but did not demonstrate a statistically significant reduction in the control group (p = 0.096).

The intragroup comparisons pointed to a statistically significant gain in CAL in both groups at 3- and 6- month evaluations (p < 0.05), and intergroup analysis revealed statistically significant differences between groups at the respective timepoints (p = 0.017 and p < 0.001, respectively) in favour of the test group. The change in CAL between 3- and 6- months was statistically significant in the test group (p = 0.003), but did not demonstrate statistically significant improvements in the control group (p = 0.084).

Regarding changes in BOP, both study groups statistically significantly improved at 3 and 6 months compared to baseline (p < 0.001). The difference between groups was statistically significant at both the 3-month (p = 0.003) and the 6-month follow-up (p < 0.001). The change between 3- and 6-month evaluation was statistically significant in the test group (p = 0.002) but not (p = 0.072) in the control group.

In terms of PI, both groups showed statistically significant improvements at 3- and 6-month follow-ups compared to baseline (p < 0.05). The intergroup comparison revealed a statistically significant difference between groups in favour of the test group at 3 (p = 0.002) and 6 months (p = 0.006). The change between 3- and 6-month evaluation was statistically significant in the test group (p = 0.003) but not (p = 0.062) in the control group.

## Discussion

Recent studies indicated that clinical outcomes of non-surgical periodontal therapy can be improved by the adjunctive subgingival application of sodium hypochlorite/amino acids and cross-linked hyaluronic acid gels.^10,25,26^ The present study investigated the microbiological impact of subgingivally delivered sodium hypochlorite/amino acids and cross-linked hyaluronic acid gels as adjuncts to same-day full-mouth subgingival debridement. To the authors’ best knowledge, this is the first study to clinically evaluate the microbiological outcomes of this novel concept (i.e., “Clean and Seal”) for non-surgical periodontal therapy.

Based on the present data, both treatment approaches (i.e., subgingival debridement and subgingival debridement in conjunction with sodium hypochlorite/amino acids and cross-linked hyaluronic acid gels) led to statistically significant microbiological shifts. However, these shifts exhibited different patterns between the test and control groups. In particular, after 3 months, both groups demonstrated statistically significant reductions in the detection frequency of T.f and T.d (p < 0.05), with the test group additionally showing a statistically significant reduction for P.i and P.g (p < 0.05). After 6 months, the detection frequency of T.f and T.d was comparable to baseline in the control group (p > 0.05), whereas statistically significant reductions (p < 0.05) compared to baseline were sustained in the test group for the respective bacterial species (T.f, T.d, P.i, and P.g). At this point, it is important to mention that the frequency of detecting A.a was unaffected by both treatment approaches (p > 0.05).

Similar findings have been reported in previous clinical studies on the effects of subgingival debridement on periodontal pathogens using molecular techniques, such as DNA probes and PCR amplification.^9,12,33^ More specifically, only the levels of P.g, T.f, T.d and P.i statistically significantly decreased after non-surgical periodontal therapy,^9,12,33^ while such changes were found to be statistically insignificant in terms of decreasing A.a.^9,33^ These findings once again support the results from previous reports which failed to demonstrate the effectiveness of subgingival debridement alone in reducing A.a. levels.^35^ Moreover, several studies have shown that statistically significant reductions in detection frequency of P.g, T.f, and T.d may be a characteristic feature of successful periodontal therapy.^7^ Our observations align well with this statement, since at 3 months, a statistically significant reduction in detection frequency was found for P.g, T.f and T.d in the test group (p < 0.05) and for T.f and T.d in the control group (p < 0.05), whereas after 6 months, the reductions remained stable for P.g, T.f and T.d only in the test group (p < 0.05).

Regarding the changes of detection scores, after 3 months, both study groups demonstrated statistically significant reductions of T.f, T.d, P.g, and P.i (p < 0.05) compared to baseline, while a statistically significant reduction of A.a was only observed in the test group (p = 0.001). At 6 months, a statistically significant reduction compared to baseline persisted for P.g and T.f in the control group (p < 0.001). However, in the test group, the reduction remained statistically significant for all investigated periodontal pathogenic species compared with baseline (p < 0.05). These findings corroborate those obtained in a recent 12-month randomised controlled clinical trial^24^ that evaluated changes in detection scores for five periodontal pathogenic species and pointed towards statistically significant benefits of the adjunctive application of sodium hypochlorite/amino acids gel to subgingival debridement in reducing the detection scores of P.g (p = 0.015) and T.f (p = 0.004). However, the levels of A.a remained unchanged compared with baseline (p = 0.098).^24^ Moreover, another clinical trial, which investigating the presence or absence of six target microorganisms in pockets treated with either ultrasonic instrumentation (control) or ultrasonic instrumentation supplemented with sodium hypochlorite/amino acid gel (test), found statistically significant reductions in T.f from baseline to day 7 (p < 0.05) and in T.d from baseline to month 4 (p < 0.05) in the test group.^19^

The differences observed in the present analysis between the test and control groups regarding detection frequencies and changes in detection scores may be attributed to the additive antimicrobial effects of sodium hypochlorite/amino acid and cross-linked hyaluronic acid gels.^17,23^ Based on previous findings from in-vitro and animal experiments, it may be hypothesised that the ability of sodium hypochlorite/amino acids to facilitate mechanical debridement and biofilm removal may lend additional support to xHyA in expressing its bacteriostatic and wound healing properties.^17,23,30-31^ In fact, as pointed out by the numerous clinical studies,^4,8,13,20^ mechanical debridement alone has only limited efficacy in eradicating all bacteria, particularly keeping in mind that bacteria may reside in soft tissues, root surface irregularities and dentinal tubules.^22^

The present work also analysed microbial samples taken from treated patients from our previous randomised clinical trial;^26^ the results are reported here. We therefore show that previously reported clinical data^26^ align well with the microbiological outcomes reported in this paper. When interpreting the data, it is important to point out that the obtained microbiological findings correspond well with the clinical outcomes assessed after 3 and 6 months after treatment. In particular, after 3 months, both study groups demonstrated statistically significant improvements in PD, BOP, PI reductions and CAL gain with a statistically significant difference in favour of the test group. An interesting finding was that after 6 months, the test group exhibited gradual and significant clinical improvements in PD, CAL, BOP, and PI compared to the 3-month evaluation. In contrast, the results in the control group remained unchanged or showed signs of relapse.

Taken together, these findings demonstrate that the microbiological benefits of sodium hypochlorite/amino acids and cross-linked hyaluronic acid gels were sustained over a 6-month period, indicating a long-term microbiological effect. Furthermore, a connection between clinical and microbiological status can be confirmed; however, it remains unclear whether a decrease in subgingival microbiota led to an improvement in clinical conditions or vice versa. When interpreting the results, the question arises as to what extent each of the adjunctive substances used (i.e., sodium hypochlorite/amino acids and cross-linked hyaluronic acid) contributed to the additional microbiological improvements observed in the test group. In this respect, it is important to emphasise that the present study used the combination of the two materials as a single concept. Therefore, further studies are needed to better understand the separate and combined effects of the two components on the clinical and microbiological outcomes.

## Conclusion

The microbiological results of the present study support the adjunctive subgingival application of sodium hypochlorite/amino acid and xHyA to subgingival debridement in the treatment of periodontitis.
